# Association between serum apolipoprotein B and atrial fibrillation: a case–control study

**DOI:** 10.1038/s41598-022-13773-2

**Published:** 2022-06-10

**Authors:** Xia Zhong, Huachen Jiao, Dongsheng Zhao, Jing Teng

**Affiliations:** 1 Department of First Clinical Medical College, Shandong University of Traditional Chinese Medicine, Jinan, Shandong People’s Republic of China; 2 Department of Cardiology, Affiliated Hospital of Shandong University of Traditional Chinese Medicine, No. 16369, Jingshi Road, Lixia District, Jinan, Shandong People’s Republic of China

**Keywords:** Biomarkers, Cardiology, Diseases, Risk factors

## Abstract

The relationship between apolipoprotein B (APOB) and atrial fibrillation (AF) is less well-known. We aimed to investigate the association between APOB and AF by gender. We conducted a case–control study including 1913 consecutive hospitalized patients to analyze the association between APOB and AF. 950 AF patients and 963 age-, sex-matched non-AF patients with sinus rhythm were evaluated. T-test, Mann–Whitney test, ANOVA, and Chi-square analysis were performed to analyze baseline data and intergroup comparisons. Pearson's correlation tests or Spearman correlation tests were performed to determine the interrelationships. Multiple regression analysis was performed to adjust for covariables. The receiver operator characteristic (ROC) curve was constructed to examine the performance of APOB. AF patients had lower APOB (P < 0.001) and an independent negative association between APOB and AF in both genders adjusting for confounding factors (OR 0.121, 95% CI 0.067–0.220, P < 0.001), regardless of statin use. APOB was positively correlated with total cholesterol (TC) (r = 0.529, p < 0.001), low-density lipoprotein cholesterol (LDL-C) (r = 0.545, p < 0.001), apolipoprotein A1 (APOA1) (r = 0.083, p < 0.001), and albumin (ALB) (r = 0.134, p < 0.001). ROC curve analysis showed that APOB level = 0.895 g/L was the most optimal cut-off value, the area under the ROC curve was 0.722. This study shows a protective association of APOB with AF in men and women. It implies APOB may be a potential biomarker for AF with a promising cut-off point of 0.895 g/L and may involve initiating and maintaining AF along with several metabolic factors.

## Introduction

Atrial fibrillation (AF) is an increasingly epidemic arrhythmia, affecting 33 million population worldwide and contributing to a rising tide of substantial morbidity, mortality, and considerable healthcare burden^1–3^. Nevertheless, the contribution of AF seems to be expected in the future, with the overall prevalence of AF estimated to triple by 2050 compared to 2006^4,5^. In addition, AF has been considered a burgeoning health threat associated with stroke, heart failure, systemic embolism, and even mortality^6–9^. Although antiarrhythmic drugs and ablation approaches as effective therapies recommended for clinical practice, the rate of hospitalization remains higher. There is increasing evidence that screening strategies for potential risk factors and comprehensive management interventions for risk factors may be beneficial in reducing the incidence of AF^10^. This leads us to focus on exploring available blood biomarkers associated with early pathological changes in AF patients and may contribute to finding several new indicators involved in AF early pathogenesis.

Currently, LDL cholesterol (LDL-C) as a predictor and therapeutic target of atherosclerotic is indisputable^11^. Apolipoprotein B (APOB) is a key structural component of LDL playing an important role in lipid metabolism^12^. Recently, APOB is also recognized as an emerging biomarker for predicting cardiovascular events, it has gradually shown a more robust and standardized predictive advantage than LDL cholesterol^11,13^. There is growing evidence that increased APOB serum levels are strongly associated with a higher risk of cardiovascular disease^14–16^. A cohort study based on 4232 individuals in the large Stockholm area reported that elevated APOB levels were related to the progression of atherosclerotic lesions and can be considered an important predictor for early cardiovascular events^17^. Previous studies have indicated that dyslipidemia may be involved in the pathogenesis of AF^18,19^. However, the effect of dyslipidemia on atrial fibrillation is still controversial. In the past, several studies have found that lower LDL cholesterol is associated with an increased risk of AF^20–22^. This discovery aroused our interest in exploring the relationship between serum APOB and AF. To the best of our knowledge, no systematic studies have been reported on the effects of APOB serum levels on AF and related metabolic factors.

The purpose of this study was to investigate the relationship between serum APOB and AF stratified by gender and explore the correlation between APOB and metabolic factors to help find some early serum biomarkers for AF.

## Material and methods

### Study design and data source

We performed a case–control study. The data of 1913 consecutive hospitalized patients (M/F: 949/964, 68.26 ± 11.02 years) who had normal dietary and exercise habits from the Affiliated Hospital of Shandong University of Traditional Chinese Medicine between January 2019 to September 2021 were collected and evaluated. Specifically, we included 950 patients aged 28–85 years and 479 men with AF, including paroxysmal AF, persistent AF, and permanent AF. 963 controls with sinus rhythm and without a history of AF were matched (at an approximately 1:1 proportion) with cases for gender and age. To better control for confounding factors, we included patients who were not receiving anticoagulant drugs at this stage of treatment because they required discontinuation or were reluctant to use anticoagulants, or were intolerant to anticoagulants. In addition, patients undergoing cardiac surgery, valvular disease, heart failure, liver and kidney dysfunction, hyperthyroidism, malignancy, use of diuretics, uric-lowering drugs, and pregnant women were excluded. Clinical characteristics of participants were investigated in an electronic medical record review, including age, sex, laboratory data, AF types, and AF complications. The data of AF patients were stratified by sex and APOB serum level. This study followed the principles of the Helsinki Declaration and was approved by the Medical Research Ethics Committee of The Affiliated Hospital of Shandong University of Traditional Chinese Medicine. Because the data are anonymized, the Ethics Committee of Affiliated Hospital of Shandong University of Traditional Chinese Medicine (NO.20200512FA62) waived the need for informed consent.

### Definition of AF

According to guidelines^23^, paroxysmal AF was defined as spontaneous AF or AF terminated by intervention within 7 days of onset. Persistent AF was defined as continuous AF sustained beyond 7 days. Permanent AF was defined as AF that sinus rhythm cannot continue to recover or maintain. AF duration was defined as the time from the first documented occurrence of AF to randomization^24^.

### APOB serum levels measurement

Serum APOAB levels were measured by immunonephelometry using a BN II analyzer (Siemens Healthcare, Marburg, Germany), and the lowest detectable concentration of APOB was 0.021 g/L.

### Screened indicators

We screened baseline data of all samples including age, gender, AF types, AF complications, and laboratory data including blood lipid profiles, aspartate aminotransferase (AST), alanine aminotransferase (ALT), prealbumin (PAB), lipoprotein (a) [Lp(a)], serum creatinine (SCr), serum uric acid (SUA), serum albumin (ALB), serum apolipoprotein A1 (APOA1), serum apolipoprotein B (APOB), as well as medication situation.

### Statistical analysis

All statistical analyses were conducted using SPSS software (version 26.0; SPSS Inc., Chicago, IL, USA). Specifically, continuous data were presented as mean ± standard deviations (SD) or medians and interquartile ranges (IQR) and compared by analysis of T-test or Mann–Whitney test and analysis of variance (ANOVA). Categorical data were expressed as percentages and compared by chi-square analysis. Meanwhile, Pearson correlation tests or Spearman correlation tests were performed to investigate interrelationships. Multivariate regression analyses were used to adjust for covariates. Additionally, the receiver operating characteristic (ROC) curve model was performed to explore the performance of the serum APOB. A two-tailed p-value < 0.05 was considered significant.

### Ethics approval and consent to participate

Ethics approval was approved by the Ethics Committee of Affiliated Hospital of Shandong University of Traditional Chinese Medicine. Because the data are anonymized, the Ethics Committee of Affiliated Hospital of Shandong University of Traditional Chinese Medicine (NO.20200512FA62) waived the need of informed consent.

## Results

### Baseline characteristics of participants

Table 1 showed the baseline characteristics of participants in two groups. We analyzed data from 1913 consecutive hospitalized patients (M/F: 949/964, 68.26 ± 11.02 years), including 950 AF patients with a duration of 24.33 (1.00–85.17) months and 963 controls (Table 1). Overall, the AF group was more likely to experience CHD, hypertension, diabetes and used drugs of β-blockers, CCBs, ACEI/ARB, and statins (p < 0.001), significantly lower levels of TG, TC, LDL-C, HDL-C, PAB, ALB, APOAl, and APOB (P < 0.05), significantly higher levels of AST, SCr, and SUA (P < 0.05). In addition, we found no significant difference in Lp (a) levels between the two groups (P > 0.05).

**Table 1:** Baseline characteristics of participants.

Variable	AF group (n = 950)	Control group (n = 963)	P value
Age, years	68.61 ± 10.34	67.92 ± 11.66	0.171
Male, n (%)	479 (50.4)	470 (48.8)	0.480
CHD, n (%)	840 (88.4)	241 (25.0)	< 0.001*
Hypertension, n (%)	638 (67.2)	324 (33.6)	< 0.001*
Diabetes, n (%)	280 (29.5)	158 (16.4)	< 0.001*
TC, mmol/L	4.19 ± 1.10	5.02 ± 1.10	< 0.001*
TG, mmol/L	1.24 ± 0.88	1.38 ± 1.24	0.004*
HDL-C, mmol/L	1.07 ± 0.30	1.21 ± 0.25	< 0.001*
LDL-C, mmol/L	2.50 ± 0.90	2.96 ± 0.86	< 0.001*
AST, U/L	20.00 (16.00–25.00)	18.00 (15.00–23.00)	< 0.001*
ALT, U/L	16.00 (12.00–24.00)	17.00 (12.00–23.00)	0.429
PAB, g/L	19.56 ± 5.94	22.26 ± 5.50	< 0.001*
Lp (a), mg/dL	23.55 ± 26.88	22.56 ± 24.34	0.399
SCr, μmoI/L	78.46 ± 51.47	64.77 ± 26.54	< 0.001*
SUA, mg/dL	5.71 ± 1.91	5.21 ± 1.50	< 0.001*
ALB, g/L	38.05 ± 4.64	40.11 ± 4.12	< 0.001*
APOA1, g/L	1.13 ± 0.26	1.23 ± 0.25	< 0.001*
APOB, g/L	0.80 ± 0.38	0.99 ± 0.24	< 0.001*
β-blockers, n (%)	743 (78.21)	159 (16.51)	< 0.001*
CCBs, n (%)	343 (36.11)	166 (17.24)	< 0.001*
ACEI/ARB, n (%)	533 (56.11)	139 (14.43)	< 0.001*
Statins, n (%)	627 (66.00)	217 (22.53)	< 0.001*
AF duration, months	24.33 (1.00–85.17)	-	-
Paroxysmal AF (155)	0.05 ± 0.05	-	-
Persistent AF (265)	4.00 (1.00–12.17)	-	-
Permanent AF (530)	73.00 (36.50–121.67)	-	-

Data were presented as mean ± SD or n (%).

*AF* atrial fibrillation, *CHD* coronary heart disease, *TC* total cholesterol, *TG* triglyceride, *LDL-C* low-density lipoprotein cholesterol, *HDL-C* high-density lipoprotein cholesterol, *AST* aspartate aminotransferase, *ALT* alanine aminotransferase, *APOA1* serum apolipoprotein A1, *APOB* serum apolipoprotein B, *ALB* serum albumin, *PAB* prealbumin, *Lp (a)* lipoprotein (a), *SCr* serum creatinine, *SUA* serum uric acid.

*Statistically significant value (P < 0.05).

**Figure 1:**
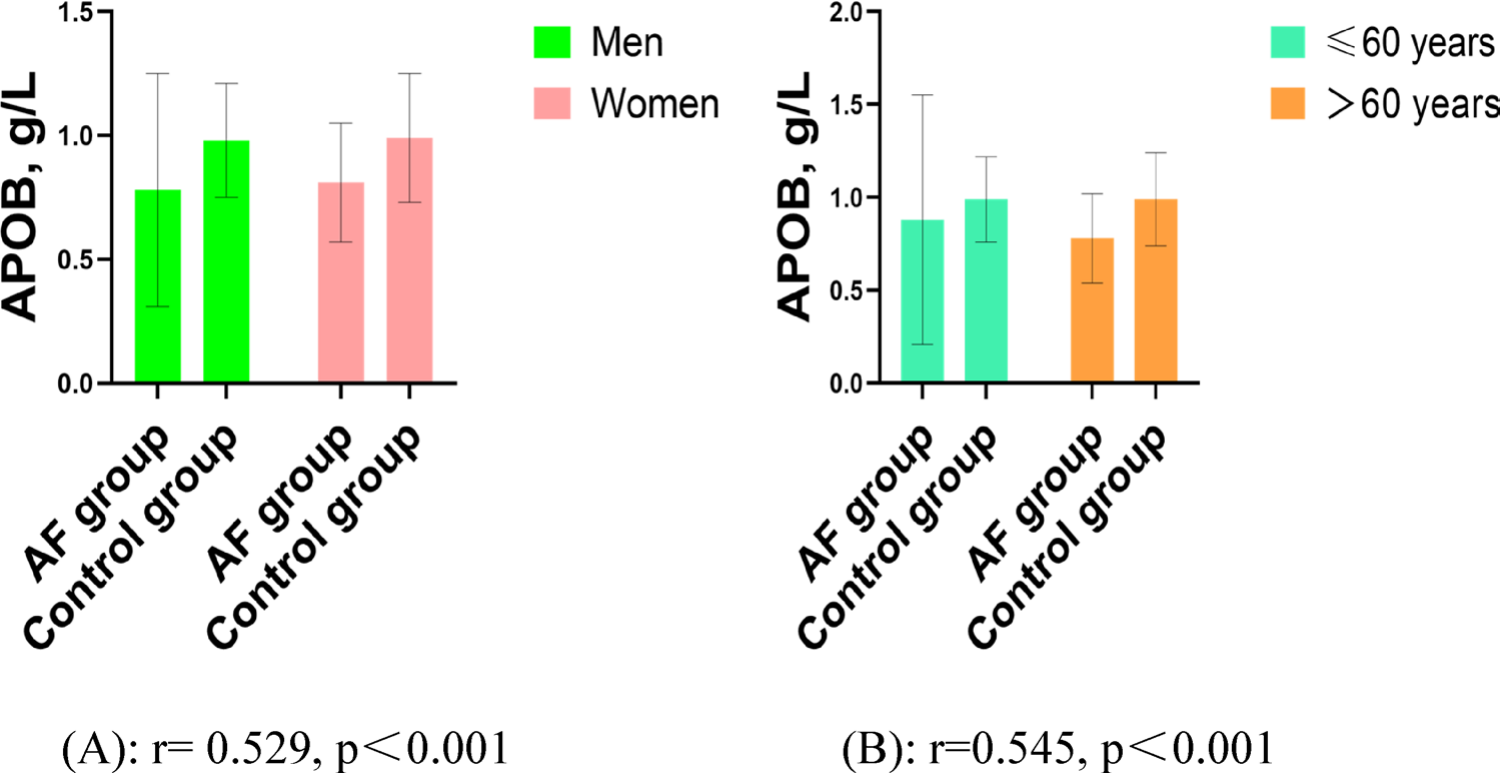
APOB levels in the AF group and control group by age and gender. Compared with controls, APOB levels of the AF group were significantly lower in the men (0.78 ± 0.47 vs. 0.98 ± 0.23 g/L, P < 0.001) and women (0.81 ± 0.24 vs. 0.99 ± 0.26 g/L, P < 0.001). Compared with controls, APOB levels of the AF group were significantly lower in the patients with age ≤ 60 years (0.88 ± 0.67 vs. 0.99 ± 0.23 g/L, P < 0.001) and age > 60 years (0.78 ± 0.24 vs. 0.99 ± 0.25 g/L, P < 0.001). Abbreviations as in Table 1. The figures were developed by GraphPad Prism software (version 9.0.0).

Figure 1 showed the difference in APOB levels between the AF group and controls by gender and age. Compared with controls, APOB levels of AF patients were significantly lower in the men (0.78 ± 0.47 vs. 0.98 ± 0.23 g/L, P < 0.001, Fig. 1A) and women (0.81 ± 0.24 vs. 0.99 ± 0.26 g/L, P < 0.001, Fig. 1A), as well as in the patients with age ≤ 60 years (0.88 ± 0.67 vs. 0.99 ± 0.23 g/L, P < 0.001, Fig. 1B) and age > 60 years (0.78 ± 0.24 vs. 0.99 ± 0.25 g/L, P < 0.001, Fig. 1B) .

### Correlation between serum APOB and AF

Table 2 showed the correlation between serum APOB and AF by multivariate regression analysis. After adjusting for CHD, hypertension, diabetes, β-blockers, CCBs, ACEI/ARB, and statins, APOB was considered to be an associated factor of AF (OR 0.096, 95% CI 0.055–0.166, P < 0.001). After adjusting for AST, SCr, SUA, ALB, APOA1, and PAB, APOB remained a significant factor related to AF (OR 0.075, 95% CI 0.048–0.119, P < 0.001). After further adjustment for all confounding factors, APOB remained an important relevant factor for AF (OR 0.121, 95% CI 0.067–0.220, P < 0.001). Moreover, the independent association was significant in both genders (P < 0.05).

**Table 2:** Correlation between serum APOB and AF.

	Total		Men		Women	
	OR 95% CI	P value	OR 95% CI	P value	OR 95% CI	P value
Model1	0.050 (0.033–0.076)	< 0.001*	0.048 (0.026–0.087)	< 0.001*	0.053 (0.030–0.093)	< 0.001*
Model2	0.096 (0.055–0.166)	< 0.001*	0.147 (0.065–0.333)	< 0.001*	0.070 (0.032–0.151)	< 0.001*
Model3	0.075 (0.048–0.119)	< 0.001*	0.103 (0.052–0.202)	< 0.001*	0.047 (0.025–0.090)	< 0.001*
Model4	0.121 (0.067–0.220)	< 0.001*	0.267 (0.106–0.669)	0.005*	0.063 (0.027–0.147)	< 0.001*

Model 1: crude, no adjustment. Model 2: adjusting for CHD, hypertension, diabetes, β-blockers, CCBs, ACEI/ARB, and statins. Model 3: adjusting for AST, SCr, SUA, ALB, APOA1, and PAB. Model 4: adjusting for all these factors. Abbreviations as in Table 1.

*Statistically significant value (P < 0.05).

### Correlation between serum APOB and AF patients with non-receiving statins

Table 3 showed the correlation between serum APOB and AF in patients with non-receiving statins by multivariate regression analysis. After adjusting for CHD, hypertension, diabetes, β-blockers, CCBs, and ACEI/ARB, APOB was associated with AF (OR 0.045, 95% CI 0.016–0.123, P < 0.001). After adjusting for AST, SCr, SUA, ALB, APOA1, and PAB, APOB remained a significant factor related to AF (OR 0.051, 95% CI 0.024–0.108, P < 0.001). After further adjustment for all confounding factors, APOB remained an important relevant factor for AF (OR 0.075, 95% CI 0.025–0.229, P < 0.001). Moreover, the independent association was significant in both sexes (P < 0.05).

**Table 3:** Correlation between serum APOB and AF in patients with non-receiving statins.

	Total		Men		Women	
	OR 95% CI	P value	OR 95% CI	P value	OR 95% CI	P value
Model1	0.024 (0.012–0.047)	< 0.001*	0.008 (0.003–0.022)	< 0.001*	0.059 (0.025–0.138)	< 0.001*
Model2	0.045 (0.016–0.123)	< 0.001*	0.024 (0.005–0.124)	< 0.001*	0.087 (0.023–0.323)	< 0.001*
Model3	0.051 (0.024–0.108)	< 0.001*	0.022 (0.006–0.075)	< 0.001*	0.069 (0.026–0.183)	< 0.001*
Model4	0.075 (0.025–0.229)	< 0.001*	0.038 (0.006–0.251)	0.001*	0.112 (0.026–0.489)	0.004*

Model 1: crude, no adjustment. Model 2: adjusting for CHD, hypertension, diabetes, β-blockers, CCBs, and ACEI/ARB. Model 3: adjusting for AST, SCr, SUA, ALB, APOA1, and PAB. Model 4: adjusting for all these factors. Abbreviations as in Table 1.

*Statistically significant value (P < 0.05).

### The ROC curve model for APOB levels predicting AF

**Figure 2:**
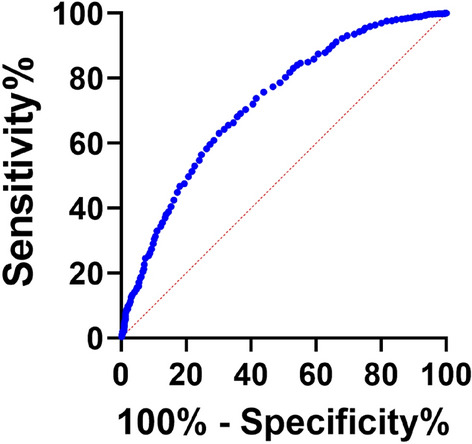
ROC curve for the APOB levels. The area under the ROC curve was 0.722 (95% CI 0.70–0.74, P < 0.05). When the most optimal cut-off value of the APOB level was 0.895 g/L, the sensitivity was 0.699, and the specificity was 0.630. Abbreviations as in Table 1. The figure was developed by GraphPad Prism software (version 9.0.0).

Figure 2 showed the ROC curve model for APOB levels predicting AF. The ROC curve analysis showed that APOB level = 0.895 g/L was the most optimal cut-off value for predicting AF. The area under the ROC curve for the model was 0.722 (95%CI: 0.70–0.74, P < 0.05), and the sensitivity was 0.699, the specificity was 0.630.

### Correlation between serum APOB and AF related factors

**Figure 3:**
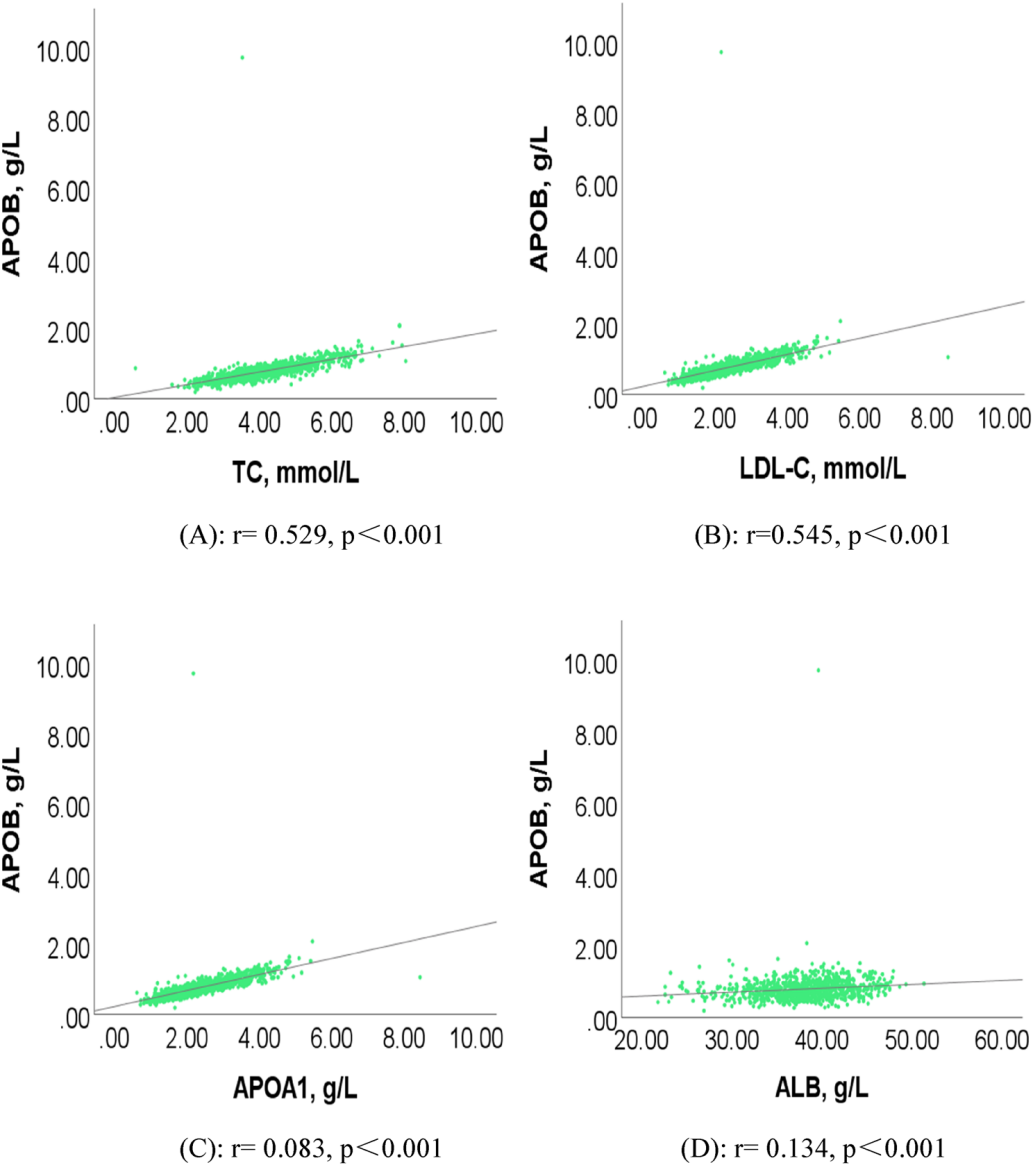
The related factors of APOB levels in AF patients. (**A**) Correlation between APOB and TC in AF group (r = 0.529, p < 0.001). (**B**) Correlation between APOB and LDL-C in AF group (r = 0.545, p < 0.001). (**C**) Correlation between APOB and APOA1 in AF group (r = 0.083, p < 0.001). (**D**) Correlation between APOB and ALB in AF group (r = 0.134, p < 0.001). Abbreviations as in Table 1. The figures were developed by SPSS software (version 26.0, SPSS Inc., Chicago, IL, USA).

Figure 3 showed the correlation between serum APOB and AF related factors. Our results suggested that APOB was positively correlated with TC (r = 0.529, p < 0.001, Fig. 3A), LDL-C (r = 0.545, p < 0.001, Fig. 3B), APOA1 (r = 0.083, p < 0.001, Fig. 3C), and ALB (r = 0.134, p < 0.001, Fig. 3D).

### Spearman correlation analysis to evaluate the association of APOB with metabolic factors by gender in AF patients

As shown in Table 4, APOB was positively correlated with PAB (r = 0.345, p < 0.001), ALB (r = 0.190, p < 0.001), SUA (r = 0.180, p < 0.001), TG (r = 0.560, p < 0.001), TC (r = 0.858, p < 0.001), and LDL-C (r = 0.888, p < 0.001) in men with patients AF. Meanwhile, APOB was positively associated with PAB (r = 0.243, p < 0.001), ALB (r = 0.253, p < 0.001), TG (r = 0.437, p < 0.001), TC (r = 0.830, p < 0.001), and LDL-C (r = 0.871, p < 0.001) in women with patients AF.

**Table 4:** Spearman correlation analysis to evaluate the association of APOB with metabolic factors by gender in AF patients.

	Men (n = 479)		Women (n = 471)	
	r	P value	r	P value
SCr, μmoI/L	0.023	0.610	− 0.038	0.408
AST, U/L	− 0.073	0.112	− 0.021	0.652
APOA1, g/L	0.129	0.005	0.104	0.024
PAB, g/L	0.345	< 0.001*	0.243	< 0.001*
ALB, g/L	0.190	< 0.001*	0.253	< 0.001*
SUA,mg/dL	0.180	< 0.001*	0.084	0.070
TG, mmol/L	0.560	< 0.001*	0.437	< 0.001*
TC, mmol/L	0.858	< 0.001*	0.830	< 0.001*
LDL-C, mmol/L	0.888	< 0.001*	0.871	< 0.001*
HDL-C, mmol/L	− 0.005	0.912	0.020	0.664

Data were presented as mean ± SD. Abbreviations as in Table 1.

*Statistically significant value (P < 0.05).

### Spearman correlation analysis to evaluate the association of APOB with metabolic factors by gender in AF patients

As shown in Table 5, patients with AF of lower APOB had lower PAB, ALB, TG, TC, and LDL-C in both sexes (P < 0.001), as well as lower APOA1 and SUA in the men (P < 0.001).

**Table 5:** One-way ANOVA for subgroups to investigate the association between APOB levels and metabolic factors in AF patients.

Variable	Men (n = 479)				Women (n = 471)			
	≤ 0.66 g/L	0.66–0.87 g/L	≥ 0.87 g/L	P value	≤ 0.66 g/L	0.66–0.87 g/L	≥ 0.87 g/L	P value
Number, n	180	148	151		142	152	177	
SCr, μmoI/L	85.22 ± 35.15	80.26 ± 18.85	82.27 ± 20.85	0.238	85.20 ± 110.14	72.57 ± 47.84	66.49 ± 20.11	0.046
AST, U/L	31.89 ± 96.12	26.26 ± 29.16	23.00 ± 15.72	0.414	24.03 ± 23.66	23.14 ± 15.86	22.61 ± 13.28	0.777
APOA1, g/L	1.05 ± 0.26	1.03 ± 0.22	1.14 ± 0.24	< 0.001*	1.16 ± 0.25	1.20 ± 0.27	1.21 ± 0.28	0.229
PAB, g/L	17.86 ± 5.50	19.31 ± 6.30	23.07 ± 6.12	< 0.001*	17.84 ± 5.37	19.01 ± 5.40	20.36 ± 5.43	< 0.001*
ALB, g/L	37.35 ± 4.26	37.43 ± 5.06	39.21 ± 4.82	< 0.001*	36.68 ± 4.66	38.43 ± 4.25	39.07 ± 4.32	< 0.001*
SUA,mg/dL	5.77 ± 1.91	5.91 ± 1.74	6.59 ± 1.90	< 0.001*	5.17 ± 2.00	5.36 ± 1.89	5.45 ± 1.71	0.403
TG, mmol/L	0.86 ± 0.51	1.18 ± 0.63	1.61 ± 1.06	< 0.001*	0.96 ± 0.41	1.25 ± 0.54	1.56 ± 1.33	< 0.001*
TC, mmol/L	3.11 ± 0.56	3.97 ± 0.56	5.12 ± 0.79	< 0.001*	3.37 ± 0.63	4.26 ± 0.59	5.32 ± 0.95	< 0.001*
HDL-C, mmol/L	1.03 ± 0.32	0.98 ± 0.24	1.03 ± 0.25	0.187	1.12 ± 0.29	1.13 ± 0.30	1.14 ± 0.34	0.851
LDL-C, mmol/L	1.64 ± 0.38	2.41 ± 0.44	3.28 ± 0.63	< 0.001*	1.71 ± 0.41	2.49 ± 0.47	3.44 ± 0.82	< 0.001*

Data were presented as mean ± SD. Abbreviations as in Table 1.

*Statistically significant value (P < 0.05).

## Discussion

This study was the first to systematically investigate the effects of serum APOB on AF and the correlations between serum APOB and metabolic factors associated with AF by gender. The main findings of the present study were a protective association between serum APOB and AF in both sexes, regardless of statin use. Further results indicated that serum APOB was positively correlated with TC, LDL-C, APOA1, and ALB. In addition, it also showed that APOB was positively correlated with PAB, ALB, TG, TC, and LDL-C in male and female patients with AF. The area under the ROC curve of APOB was 0.722, the most optimal cut-off value was 0.895 g/L, the sensitivity was 0.699, and the specificity was 0.630. These findings suggested that the level of APOB and some related metabolic factors may decrease in the preclinical stage of AF.

The effects of dyslipidemia on AF have been controversial. Although increased TC and LDL-C were recognized as the significant risk factors for AF^25,26^, several scholars also offered a contrary opinion^27,28^, which was called the “cholesterol paradox”. In recent years, a growing body of data from studies has reported that low levels of LDL-C were strongly associated with an increased risk of AF^29,30^. Our results showed lower levels of TG, TC, LDL-C, and HDL-C in AF patients, which were well in alignment with earlier findings.

APOB, an important element in LDL and a precursor of atherosclerosis, reflects the number of lipoprotein particles that may induce atherosclerosis^31^.To our knowledge, few studies have reported the relationship between serum APOB and AF. Fortunately, we found several previous studies that reported relevant results. Two Mendelian randomization (MR) analyses indicated no significant causal effects of serum APOB on the risk of AF^32,33^. This result was inconsistent with our findings, there are several possible reasons. First, the main possible reason is racial differences; our patients were from China, while their study population was European populations, which contributes to heterogeneity in the study population. Second, the influence of several drugs was not evaluated in their MR study. Additionally, the study methods are significantly different, possibly related to the choice of covariates included in the models. Another nested cohort study suggested that low serum APOB was the main determinant of incident AF in both genders [RR 0.44, 95% CI 0.30–0.66]^34^. Our result was consistent with this study, which showed an independent negative association between serum APOB and AF in both sexes. In addition, we built the ROC model for APOB levels predicting AF. To our knowledge, there are no reports of APOB levels predicting AF. Our results indicated the area under the ROC curve was 0.722; when the most optimal cut-off value of the APOB level was 0.895 g/L, the sensitivity was 0.699, and the specificity was 0.630. Certainly, the current result requires further verification.

Indeed, several underlying mechanisms may be considered to explain this interesting finding. Firstly, it has been well established that the effects of inflammation and oxidation stress on the complexity of AF^35–37^. Although most studies have suggested that there was almost no correlation between lipids and inflammatory markers^38–40^, several scholars still examined the association between APOB and inflammation. Faraj M et al. reported that APOB was a strong and independent predictor of several inflammatory markers such as interleukin-6 and CRP in postmenopausal overweight and obese women^41^. Further research indicated that reduced serum APOB was closely related to inflammation and increased serum APOB may be the key therapeutic target to reduce obesity-related inflammation^42^. Therefore, it could be speculated that reduced APOB may initiate and maintain the inflammatory chain of AF. Secondly, the current results showed the lower levels of HDL-C in AF participants. Thus, we hypothesized that the loss of anti-inflammatory and antioxidant effects of HDL-C increased the formation of AF matrix^43–45^, and may contribute to the formation of AF risk factors such as heart failure^14,46^. Meanwhile, potential confounding factors such as statin use, lifestyle, and dietary factors may also confound the results. Consequently, it is essential to conduct further studies to explore the potential mechanisms.

We observed several AF-related confounders and adjusted for them in the regression analysis model. Current results indicated that AST levels were higher in AF patients. In fact, the relationship between liver enzymes and AF is unclear. Sinner et al.^47^ reported transaminase concentrations are related to the increased risk of AF. The results of our study are supported by their study reporting higher AST levels in AF patients. Possible reasons mainly include preclinical heart failure^48,49^, metabolic syndrome, inflammation, oxidative stress, nonalcoholic fatty liver disease, strenuous exercise, overwork, drinking, greasy diet, irregular work and rest, and anger^50–53^. The current results also showed AF patients have more comorbidities including hypertension, coronary heart disease, and diabetes. Previous studies have demonstrated hypertension, coronary heart disease, and diabetes are associated with a higher risk of AF^54–56^. Moreover, these comorbidities are likely to form a vicious cycle with AF. Therefore, it would be interesting to investigate the association between APOB and lone AF in the future.

Additionally, we also paid attention to the correlation between serum APOB and AF-related factors. The findings indicated that serum APOB was positively correlated with TC, LDL-C, APOA1, and ALB. On this basis, we further investigated the potential relationship between APOB level and metabolic factors in men and women with AF by Spearman correlation analysis and One-way ANOVA for subgroups. We observed that APOB was positively correlated with PAB, ALB, TG, TC, and LDL-C in male and female patients with AF. These findings imply that serum APOB may be affected by PAB, ALB, TG, TC, and LDL-C, and participate in the pathological process of AF together.

Certainly, there might be some potential limitations worth considering. First, this was a single-center case–control study and this protective association outcome can’t confirm causality. Second, we did not examine indicators of inflammation and oxidative stress. Third, medication characteristics and comorbidities of the AF patients and controls were not well matched; it would be interesting to investigate the association between APOB and AF based on patients with matched medication and comorbidities in the future. Fourth, several potential confounding factors such as genetic factors, lifestyle, medication, and family history may also have influenced the current results. Nevertheless, it did provide us with a new perspective to find the potential mechanisms of AF. Further prospective longitudinal cohort studies are encouraged to be conducted. In addition, the correlation between serum APOB and AF-related metabolic factors is still worthy of further studies, which will be useful to further clarify the relationship between serum APOB and AF.

## Conclusions

In conclusion, we systematically investigated the association between serum APOB and AF. The present results indicated a protective association between serum APOB and AF in both sexes, regardless of statin use. Further findings showed that serum APOB was positively correlated with TC, LDL-C, APOA1, and ALB. These findings suggested serum APOB may be a potential biomarker for AF with a promising cut-off point of 0.895 g/L and may involve in the pathological progress of AF along with several metabolic factors. If a causal relationship between APOB and AF is confirmed, modulating APOB levels may contribute to the prevention or treatment of AF.

## Data Availability

The datasets are not publicly available due to them containing information that could compromise research participant privacy, but the minimal data are available from the corresponding author on reasonable request.
